# Associations between hyperuricemia and ultrasound-detected knee synovial abnormalities in middle-aged and older population: a cross-sectional study

**DOI:** 10.1186/s13018-024-04708-w

**Published:** 2024-04-04

**Authors:** Qianlin Weng, Ting Jiang, Weiya Zhang, Michael Doherty, Zidan Yang, Jie Wei

**Affiliations:** 1 Department of Orthopaedics, Xiangya Hospital, Central South University, Changsha, China; 2 Department of Ultrasonography, Xiangya Hospital, Central South University, Changsha, China; 3 Academic Rheumatology, School of Medicine, University of Nottingham, Nottingham, UK; 4 Pain Centre Versus Arthritis UK, University of Nottingham, Nottingham, UK; 5 Key Laboratory of Aging-related Bone and Joint Diseases Prevention and Treatment, Ministry of Education, Xiangya Hospital, Central South University, Changsha, China; 6 Hunan Key Laboratory of Joint Degeneration and Injury, Xiangya Hospital, Central South University, Changsha, China; 7 Department of Epidemiology and Health Statistics, Xiangya School of Public Health, Central South University, Changsha, China

**Keywords:** Hyperuricemia, Synovial abnormality, Knee, Ultrasound

## Abstract

**Objectives:**

Knee synovial abnormalities, potentially treatment targets for knee pain and osteoarthritis, are common in middle-aged and older population, but its etiology remains unclear. We examined the associations between hyperuricemia and knee synovial abnormalities detected by ultrasound in a general population sample.

**Methods:**

Participants aged ≥ 50 years were from a community-based observational study. Hyperuricemia was defined as serum urate (SU) level > 416 µmol/L in men and > 357 µmol/L in women. Ultrasound of both knees was performed to determine the presence of synovial abnormalities, i.e., synovial hypertrophy, effusion, or Power Doppler signal (PDS). We examined the relation of hyperuricemia to prevalence of knee synovial abnormalities and its laterality, and the dose-response relationships between SU levels and the prevalence of knee synovial abnormalities.

**Results:**

In total, 3,405 participants were included in the analysis. Hyperuricemia was associated with higher prevalence of knee synovial abnormality (adjusted odds ratio [aOR] = 1.21, 95% confidence interval [CI]: 1.02 to 1.43), synovial hypertrophy (aOR = 1.33, 95% CI: 1.05 to 1.68), and effusion (aOR = 1.21, 95% CI: 1.02 to 1.44), respectively. There were dose-response relationships between SU levels and synovial abnormalities. Additionally, the hyperuricemia was more associated with prevalence of bilateral than with that of unilateral knee synovial abnormality, synovial hypertrophy, or effusion; however, no significant association was observed between hyperuricemia and PDS.

**Conclusion:**

In this population-based study we found that hyperuricemia was associated with higher prevalence of knee synovial abnormality, synovial hypertrophy and effusion, suggesting that hyperuricemia may play a role in pathogenesis of knee synovial abnormalities.

**Supplementary Information:**

The online version contains supplementary material available at 10.1186/s13018-024-04708-w.

## Introduction

Knee synovial abnormalities are commonly found in the middle-aged and older population with a prevalence ranging from 18.1–53.1% for synovial hypertrophy, 46.6–69.7% for effusion and 4.9–31.8% for Power Doppler signal (PDS), respectively [1–3]. There is increasing evidence that synovial abnormalities are related to both knee pain and the incidence and progression of knee osteoarthritis, pointing to targets for prevention and treatment [3–8]. However, the etiology of knee synovial abnormalities remains unclear, resulting in limited intervention approaches.

Hyperuricemia is a metabolic disorder caused by abnormal serum urate (SU) metabolism [9]. Elevated SU can result in the deposition of monosodium urate (MSU) crystals and the presence of systemic inflammation, both of which may lead to knee synovial abnormalities [10–12]. To date, only a few studies have evaluated the associations between hyperuricemia and knee synovial abnormalities, the results, however, are inconsistent [13–15]. The discrepancy of these findings could partially be explained by a small sample size (ranging from 71 to 150 participants), residual confounding (i.e., without adjustment for age, sex and body mass index [BMI]), or selection bias by restricting study participants with a specific diseases (i.e., rheumatoid arthritis [RA] or osteoarthritis) [13–15]. Consequently, lack of valid epidemiological evidence makes it difficult to fully interpret the pathogenesis effect of SU on knee synovial abnormalities and may prevent examination of urate-lowering therapy as a potentially beneficial adjunctive therapy for synovial abnormalities [16, 17]. This could further hinder the future treatments for knee osteoarthritis, although currently dry needling combined with exercise, manual therapy, and pain education have been proven effective [18–20].

To help fill this knowledge gap, we conducted this cross-sectional study to investigate the associations between hyperuricemia and ultrasound detected synovial abnormalities in a large middle-aged and older general population.

## Methods

### Study design and population

Participants in the present study were from the Xiangya Osteoarthritis Study (XO Study), an ongoing community-based longitudinal study conducted in Longshan County, Hunan Province, China (NCT04033757) [3, 21–23]. The XO Study consists of three sub-cohorts (i.e., sub-cohorts I, II and III) which were initiated in 2015, 2018, and 2019, respectively. Among 46 communities included in Longshan County, we firstly adopted a probability proportionate to size (PPS) sampling method to select fourteen communities. The PPS is a method of sampling from a finite population in which a size measure is available for each population unit before sampling and where the probability of selecting a unit is proportional to its size. Subsequently, we compiled a randomized list of all villages to select the communities. Starting with the initial village in the first community, we invited the all residents aged 50 or above to take part in the study. The village-by-village recruitment process persisted until the number of participants in that community matched the pre-determined quota (i.e., the same age and sex distribution of the source population) according to the Sixth National Census Data of Longshan County (2010). A total of 25 rural mountainous villages of Longshan County were eventually included in the XO Study. Knee ultrasound assessment was introduced into the XO Study from 2017. The current study included individuals who were potentially eligible for knee ultrasound assessment in 2017 (the second-year follow-up of sub-cohort I), 2018 (the baseline of sub-cohort II) and 2019 (the baseline of sub-cohort III).

The XO study was approved by the Research Ethics Committee of Xiangya Hospital, Central South University (201510506), and all participants gave informed written consent for their participation. The protocol of the study was registered online (NCT04033757).

### Assessment of ultrasound

Ultrasound assessment was performed by a single trained sonographer (TJ), using a real-time scanner (Philips CX30) with a multi-frequency (4–12 MHz) linear transducer. The Power Doppler examination was conducted with a 400 Hz pulse repetition frequency. For each examination, a generous amounts of gel were applied to the knee and the sonographer took care in applying only minimal pressure to the transducer during the examination. The sonographer was blinded to the clinical and laboratory findings.

The suprapatellar recess was scanned in each knee in 30º flexion according to the Outcome Measures in Rheumatology (OMERACT) atlas [24]. Based on OMERACT-7 definitions [25] (Supplementary Fig. 1), the maximal thickness of synovial hypertrophy and depth of effusion were assessed in millimeters along the longitudinal axis [3]. Synovial hypertrophy was defined as a synovial thickness ≥ 4 mm, and the presence of effusion was determined when the effusion depth was ≥ 4 mm, according to the criteria established by the European League Against Rheumatism (EULAR) study [26]. The presence of PDS in the synovial membrane was observed in both longitudinal and transverse planes and dichotomized as absent or present (Supplementary Fig. 2). Participants were categorized as exhibiting synovial hypertrophy, effusion or PDS if any of these features were observed in either knee. We further classified participants into either unilateral synovial hypertrophy, effusion or PDS if only one knee had these abnormalities, or bilateral synovial hypertrophy, effusion or PDS if both knees had one or more of these features.

The intra-observer (TJ) reliability for ultrasound measures was evaluated by scoring the same ultrasound images (30 grey scale and 30 Power Doppler stored ultrasound images selected from 55 participants to show a range of severity of synovial abnormalities) on two occasions 12-week apart. To evaluate inter-observer reliability, two assessors (TJ and MH) independently reviewed the same 30 greyscale and 30 Power Doppler ultrasound images. Intra-class correlation coefficient (ICC) and weighted kappa statistic were used for continuous data and categorical data, respectively. The intra- and inter-rater reliability were high for synovial hypertrophy (range of ICC: 0.94 to 0.99), effusion (range of ICC: 0.96 to 0.98) and PDS (range of weighted Kappa statistics: 0.82 to 1.00) [3] (Supplementary Table 1).

### Assessment of hyperuricemia

All blood samples were taken in the morning after at least 12 h of fasting and were stored at 4 °C until analysis. Blood was aspirated into a Vacutainer tube containing ethylenediaminetetraacetic acid and allowed to clot before being centrifuged at 3,000 rpm for 15 min for serum separation. SU and serum creatinine were analyzed at the Clinical Laboratory of Xiangya Hospital and detected on a Beckman Coulter AU 5800 (Beckman Coulter Inc., Brea, CA, USA). The inter- and intra-assay coefficients of variation for SU were tested for low (118 µmol/L) and high concentrations (472 µmol/L). The intra-assay coefficients of variation were 1.39% (118 µmol/L) and 0.41% (472 µmol/L), respectively, and inter-assay coefficients of variation were 1.40% (118 µmol/L) and 1.23% (472 µmol/L), respectively. We defined a participant as having hyperuricemia if the SU level was > 416 µmol/L (7.0 mg/dL) in men and > 357 µmol/L (6.0 mg/dL) in women [27].

### Assessment of other covariates

Data were collected by the trained health professional researchers using standard questionnaires through a face-to-face interview. Age, sex, alcohol consumption, smoking status, history of knee injury, educational level, diabetes and hypertension status were documented. Height and weight were assessed, and BMI was determined by dividing weight (in kilograms) by square of height (in meters^2^). Blood pressure was checked on an electronic sphygmomanometer. The blood fasting glucose was also detected on a Beckman Coulter AU 5800 (Beckman Coulter Inc., Brea, CA, USA). Diabetes was diagnosed as fasting glucose level ≥ 7.0 mmol/L or if the participant was receiving drug treatment to control blood glucose [28]. Hypertension was defined as systolic blood pressure ≥ 140 mmHg or diastolic blood pressure ≥ 90 mmHg, or if the participant was taking antihypertensive treatment [29]. Gout was defined as a self-report of gout or urate-lowering therapy use [30].

### Statistical analysis

Continuous variables were presented as mean ± standard deviation (SD) and categorical variables were expressed as percentage. We calculated the prevalence of each ultrasound-detected synovial abnormality for participants according to hyperuricemia status. We examined the association of hyperuricemia with synovial abnormality using generalized estimating equations (GEE) with logit link. We obtained both crude odds ratio (OR) and multivariable adjusted OR (aOR) from GEE model and their 95% confidence interval (CI). In the multivariable adjusted regression model, we adjusted for age (< 60 years, 60–69 years, ≥ 70 years), sex (male, female), BMI (continuous variable), smoking status (never, past, current), alcohol consumption (never, past, current), educational level (educated, non-educated), knee injury history (yes, no), diabetes (yes, no), hypertension (yes, no) and serum creatinine (continuous data). The dose-response relationship between SU and the prevalence of knee synovial abnormalities was evaluated by restricted cubic splines regression with two knots defined by the tertile distribution of SU [22]. In addition, we evaluated the association of hyperuricemia with the laterality of each ultrasound-detected knee synovial abnormality using proportional odds logistic regression model. We performed a sensitivity analysis to examine the associations between asymptomatic hyperuricemia and the prevalent knee synovial abnormalities by excluding gout patients. Statistical analyses were conducted using SAS V.9.4 (SAS Institute, Cary, North Carolina, USA). All *P* values were 2-sided and *P* < 0.05 was considered significant.

## Results

**Fig. 1:**
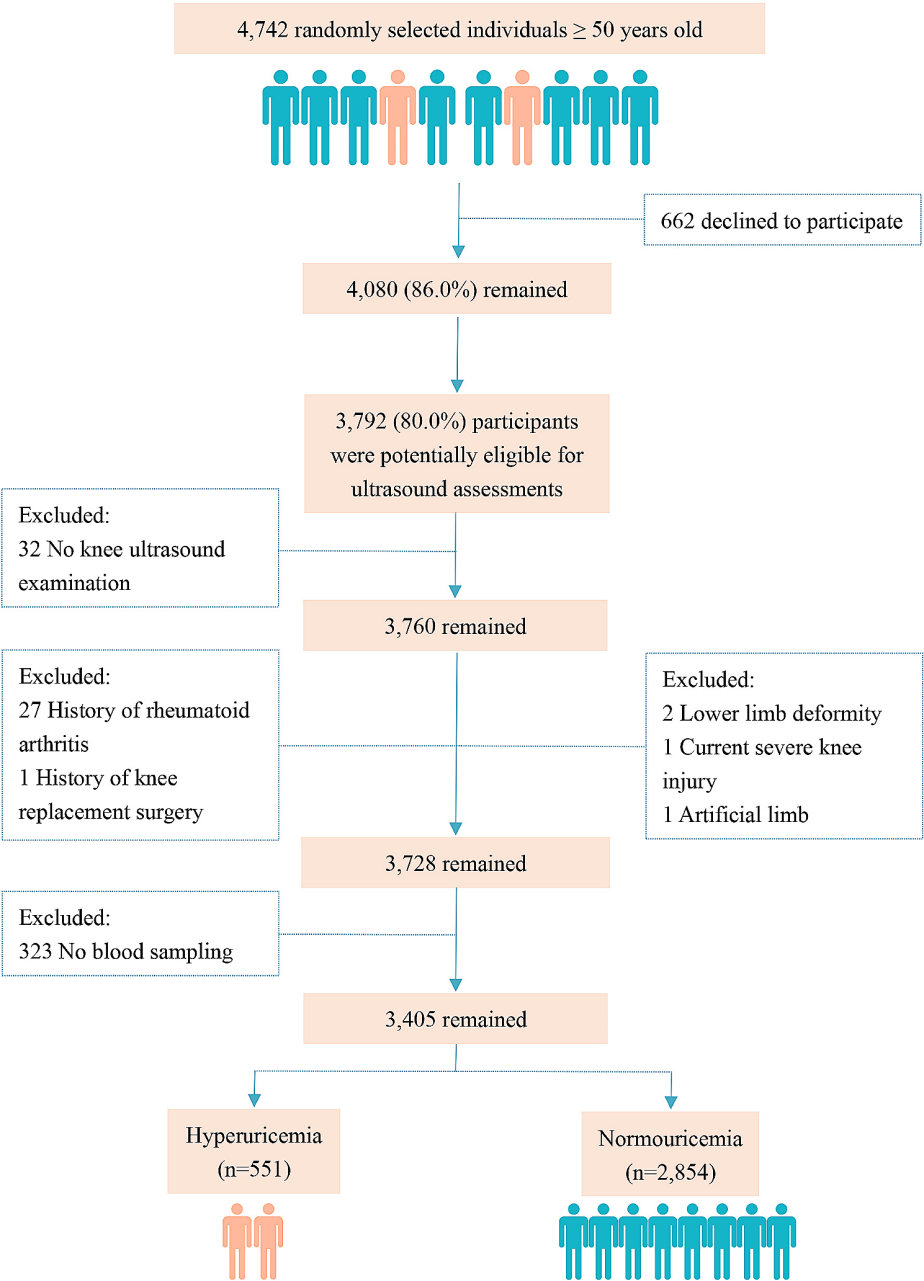
Flow chart of participants in the study

Of 3,792 participants from the XO study (Year 2017: *n* = 1181; Year 2018: *n* = 1271; Year 2019: *n* = 1340), we excluded 32 participants who did not undergo ultrasound examination and 323 who did not provide blood samples. In addition, we excluded participants with a history of RA (*n* = 27), severe lower limb deformity (*n* = 2), artificial limb (*n* = 1), current severe knee injury (*n* = 1) and previous knee replacement surgery (*n* = 1). The final sample consisted of 3,405 participants (1,465 men and 1,940 women). A flow chart of the selection of study participants is shown in Fig. 1. The characteristics of the participants are showed in Table 1. The mean age of the whole study sample was 64.5 ± 9.3 years, mean BMI was 24.0 ± 3.5 kg/m^2^, and the prevalence of hyperuricemia was 16.2% (*n* = 551) in the present study.

**Table 1:** Characteristics of the study sample for knee synovial abnormalities on ultrasound

	Total (*n* = 3,405)	Hyperuricemia (*n* = 551)	Normouricemia (*n* = 2,854)
**Women, n (%)**	1,940 (57.0)	272 (49.4)	1,668 (58.4)
**Age, years (mean ± SD)**	64.5 ± 9.3	66.9 ± 9.8	64.0 ± 9.1
50–59	33.6	26.9	34.9
60–69	35.9	33.2	36.4
≥70	30.5	39.9	28.7
**Height, cm (mean ± SD)**	151.1 ± 8.0	151.7 ± 8.4	151.0 ± 7.9
**Weight, kg (mean ± SD)**	54.9 ± 10.0	56.6 ± 11.6	54.6 ± 9.6
**BMI, kg/m** ^**2**^ **(mean ± SD)**	24.0 ± 3.5	24.5 ± 3.9	23.9 ± 3.5
**Smoking status (%)**			
Non-smoker	64.8	58.5	66.0
Ex-smoker	4.6	7.6	4.0
Current smoker	30.6	33.9	30.0
**Alcohol drinking (%)**			
Non-drinker	53.5	46.3	54.9
Ex-drinker	11.5	17.2	10.4
Current drinker	35.0	36.5	34.7
Education (educated, %)*	67.6	67.9	67.6
**Knee injury history (%)** ^†^	3.1	3.0	3.1
**Diabetes (%)**	8.8	12.9	8.0
**Hypertension (%)**	58.8	69.9	56.6
**Serum creatinine (µmol/L)**	57.9 ± 32.4	72.2 ± 39.4	55.1 ± 30.2

BMI, body mass index; n, number; SD, standard deviation

* Educated was defined as primary school or above

^†^ Knee injury history was defined as history of knee injury severely restricting walking for at least one week

The associations between hyperuricemia and the prevalence of knee synovial abnormalities are summarized in Table 2. The knee-based prevalence of synovial abnormality (i.e., synovial hypertrophy, effusion or PDS), synovial hypertrophy, effusion and PDS was 36.8%, 14.0%, 34.6% and 4.2%, respectively, in the participants with hyperuricemia, compared with 34.4%, 10.3%, 32.3% and 2.5%, respectively, in the normouricemic participants. After adjusting for potential confounders, compared with normouricemia, the aOR of knee synovial abnormality was 1.21 (95% CI: 1.02 to 1.43) for hyperuricemia. The corresponding ORs were 1.33 (95% CI: 1.05 to 1.68) for synovial hypertrophy and 1.21 (95% CI: 1.02 to 1.44) for effusion, respectively. Participants with hyperuricemia also had a higher, albeit non-statistically significant, prevalence of PDS than those without hyperuricemia (aOR = 1.40, 95% CI: 0.92 to 2.13).

**Table 2:** Associations between hyperuricemia and knee synovial abnormalities on ultrasound

Synovial abnormalities	Hyperuricemia	
	No	Yes
Knee synovial abnormality*		
No, number of knees (%)	3,747 (65.6)	696 (63.2)
Yes, number of knees (%)	1,961 (34.4)	406 (36.8)
Crude OR (95% CI)	1.00 (reference)	1.11 (0.95, 1.30)
Adjusted OR (95% CI)^†^	1.00 (reference)	1.21 (1.02, 1.43)
**Synovial hypertrophy**		
No, number of knees (%)	5,118 (89.7)	948 (86.0)
Yes, number of knees (%)	590 (10.3)	154 (14.0)
Crude OR (95% CI)	1.00 (reference)	1.41 (1.13, 1.75)
Adjusted OR (95% CI)^†^	1.00 (reference)	1.33 (1.05, 1.68)
**Joint effusion**		
No, number of knees (%)	3,865 (67.7)	721 (65.4)
Yes, number of knees (%)	1,843 (32.3)	381 (34.6)
Crude OR (95% CI)	1.00 (reference)	1.11 (0.94, 1.30)
Adjusted OR (95% CI)^†^	1.00 (reference)	1.21 (1.02, 1.44)
**Power Doppler signal**		
No, number of knees (%)	5,564 (97.5)	1,056 (95.8)
Yes, number of knees (%)	144 (2.5)	46 (4.2)
Crude OR (95% CI)	1.00 (reference)	1.68 (1.16, 2.45)
Adjusted OR (95% CI)^†^	1.00 (reference)	1.40 (0.92, 2.13)

CI, confidence interval; OR, odds ratio

* Synovial hypertrophy, joint effusion, or Power Doppler signal

^†^ Adjusted for age, sex, BMI, smoking status, alcohol consumption, educational level, knee injury history, diabetes, hypertension, and serum creatinine

**Fig. 2:**
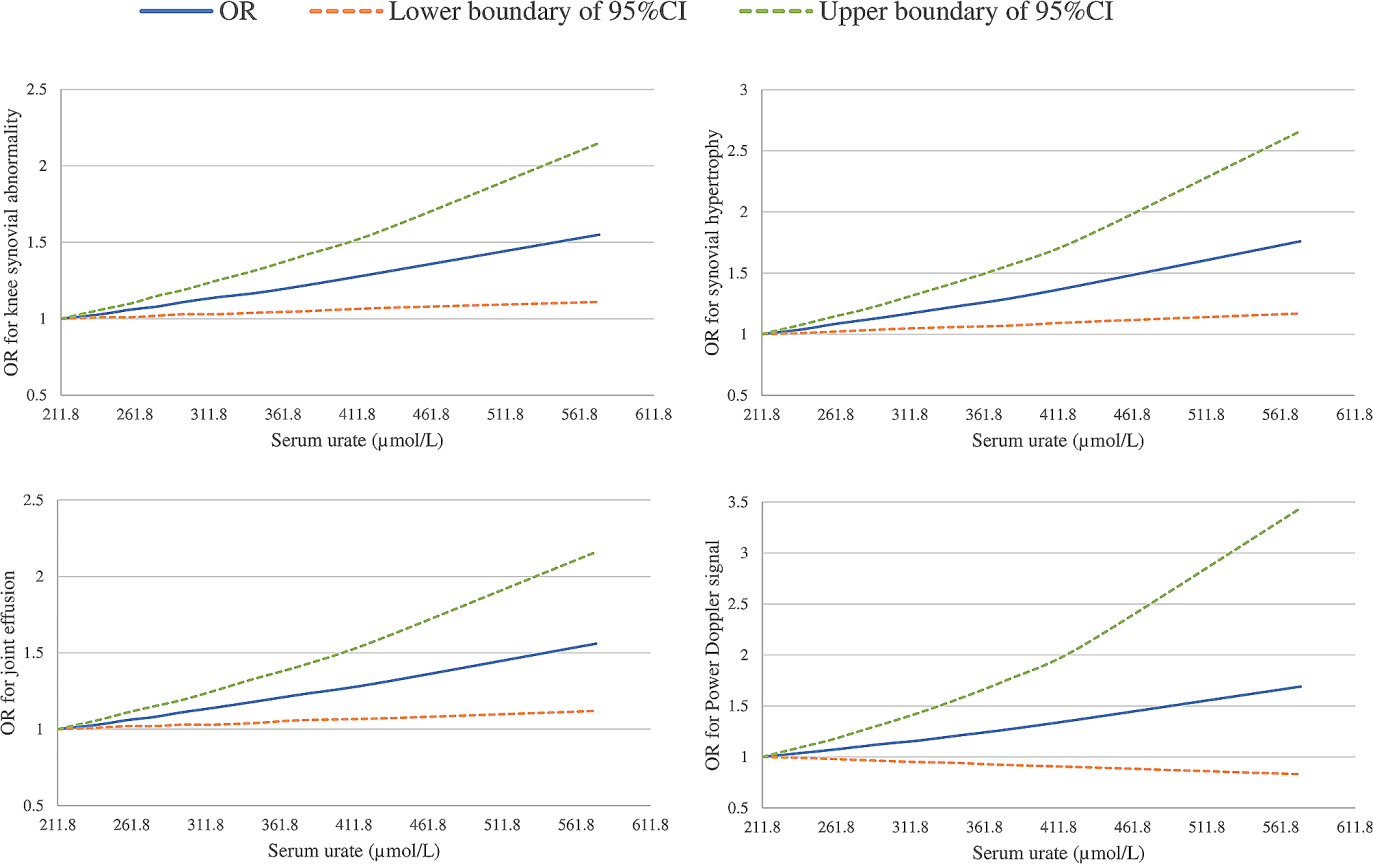
Dose-response relationship between serum urate level and the OR for knee synovial abnormalities on ultrasound. CI, confidence interval; Odds ratios were adjusted for age, sex, body mass index, smoking status, alcohol consumption, educational level, knee injury history, diabetes, hypertension, and serum creatinine

As shown in Fig. 2, SU, even within the normal range, was associated with the OR for prevalence of knee synovial abnormalities in a dose-response-relationship manner (test for trend *P* = 0.009 for knee synovial abnormality; *P* = 0.007 for synovial hypertrophy; *P* = 0.008 for effusion).

The associations of hyperuricemia with the laterality of each ultrasound-detected knee synovial abnormality are presented in Table 3. The prevalence of bilateral and unilateral knee synovial abnormality was 22.3% and 29.0% in participants with hyperuricemia, compared with 20.4% and 27.9% in those with normouricemia. The corresponding aOR was 1.24 (95% CI: 1.03 to 1.50). Specifically, we observed higher prevalence of bilateral (5.8%) and unilateral (16.3%) synovial hypertrophy in participants with hyperuricemia, compared with those with normouricemia (bilateral: 4.0%; unilateral: 12.7%), with aOR being 1.35 (95% CI: 1.05 to 1.73). Similarly, the prevalence of bilateral and unilateral effusion were 20.5% and 28.1% in participants with hyperuricemia compared with 18.4% and 27.7% in the participants with normauricemia. The aOR was 1.25 (95% CI: 1.03 to 1.51). However, the prevalence of bilateral and unilateral PDS were low (1.1% and 6.2% in the hyperuricemia participants and 0.6% and 3.8% in the normouricemia participants, respectively). The corresponding aOR was 1.42 (95% CI: 0.94 to 2.16). The results of sensitivity analyses did not change materially when we evaluated the associations between asymptomatic hyperuricemia and the prevalent knee synovial abnormalities (*n* = 42, Supplementary Tables 2 and 3).

**Table 3:** Associations of hyperuricemia and laterality of prevalent of knee synovial abnormalities on ultrasound

Synovial abnormalities	Hyperuricemia	
	No	Yes
Knee synovial abnormality*		
No, n (%)	1,476 (51.7)	268 (48.6)
Unilateral, n (%)	795 (27.9)	160 (29.0)
Bilateral, n (%)	583 (20.4)	123 (22.3)
Crude OR (95% CI)	1.00 (reference)	1.13 (0.95, 1.34)
Adjusted OR (95% CI)^†^	1.00 (reference)	1.24 (1.03, 1.50)
**Synovial hypertrophy**		
No, n (%)	2,378 (83.3)	429 (77.9)
Unilateral, n (%)	362 (12.7)	90 (16.3)
Bilateral, n (%)	114 (4.0)	32 (5.8)
Crude OR (95% CI)	1.00 (reference)	1.42 (1.14, 1.78)
Adjusted OR (95% CI)^†^	1.00 (reference)	1.35 (1.05, 1.73)
**Joint effusion**		
No, n (%)	1,537 (53.9)	283 (51.4)
Unilateral, n (%)	791 (27.7)	155 (28.1)
Bilateral, n (%)	526 (18.4)	113 (20.5)
Crude OR (95% CI)	1.00 (reference)	1.12 (0.94, 1.33)
Adjusted OR (95% CI)^†^	1.00 (reference)	1.25 (1.03, 1.51)
**Power Doppler signal**		
No, n (%)	2,728 (95.6)	511 (92.7)
Unilateral, n (%)	108 (3.8)	34 (6.2)
Bilateral, n (%)	18 (0.6)	6 (1.1)
Crude OR (95% CI)	1.00 (reference)	1.70 (1.17, 2.45)
Adjusted OR (95% CI)^†^	1.00 (reference)	1.42 (0.94, 2.16)

CI, confidence interval; n, number; OR, odds ratio

* Synovial hypertrophy, joint effusion, or Power Doppler signal

^†^ Adjusted for age, sex, BMI, smoking status, alcohol consumption, educational level, knee injury history, diabetes, hypertension, and serum creatinine

## Discussion

In this large community-based study, we found that hyperuricemia was positively associated with knee synovial abnormality, synovial hypertrophy, and effusion. The association was graded upon number of joints affected from none, unilateral to bilateral synovial hypertrophy or effusion. The SU was associated with the OR for prevalence of knee synovial abnormalities in a dose-response relationship manner. These findings support the consideration of hyperuricemia as an emerging systemic risk factor for knee synovial abnormalities.

## Comparisons with previous studies

To date, only a few studies have investigated the relationship between hyperuricemia and knee synovial abnormalities, with conflicting results [13–15]. One case-control study reported that hyperuricemia was not associated with the presence of the knee synovial abnormalities [13]. However, the authors acknowledged that the small sample size of their study (*n* = 102) might have compromised their statistical power to detect a difference between the two groups, and unbalanced demographic characteristics such as age in the two groups might also have caused bias. Conversely, in another case-control study of people with knee osteoarthritis (*n* = 71), those with a high SU level (≥ 360 µmol/L) were more likely to have knee synovitis detected by Magnetic Resonance Imaging [14]. Similarly, a cross-sectional study conducted among individuals with RA (*n* = 150) also reported higher prevalence and more severe ultrasound-detected synovial hypertrophy and effusion in multiple joints in those with hyperuricemia than to those with normouricemia [15]. However, restricting study participants to those with RA may dilute the association between SU and synovial abnormality because RA may occur after hyperuricemia and synovial abnormality and potentially cause selection bias (i.e., collider bias). In contrast, our study provided empirical evidence of the positive associations between hyperuricemia and knee synovial abnormalities in a large general population sample. Our findings are independent of potential confounders and consistent across the different measures of synovial abnormalities.

### Possible explanations

Several explanations may account for the relationship between hyperuricemia and knee synovial abnormalities. Previous studies have suggested that hyperuricemia was associated with presence of low-grade systemic inflammation even in the absence of clinical features of gout [31]. Elevated SU stimulates the pro-inflammatory response and activates mononuclear cells to increase production of interleukin-1beta (IL-1β), interleukin-6 (IL-6), tumor necrosis factor-α (TNF-α), and proinflammatory proteins [10, 32, 33]. Furthermore, systemic inflammation may exist long before inflammatory lesions are established in the synovial membrane, and several studies have shown that knee synovial abnormalities are related to systemic inflammation [34–36]. Additionally, formation of MSU crystals in and around peripheral joints may locally induce leukocytes production of cytokines, enhance the inflammation response, and elicit synovial inflammation, and thus have a local effect on pathogenic mechanisms of inflamed synovium [37–40].

### Strengths and limitations

Several strengths of this study are noteworthy. First, our study was relatively large and has an adequate power to test the research hypothesis [41]. Second, XO study is a population-based study, thus the findings may be generalizable to the general population with similar characteristics [21]. Third, we adjusted for several important confounding variables (age, sex, BMI, smoking status, alcohol consumption, educational level, previous knee injury, diabetes, hypertension, and serum creatinine) in multivariable adjusted models, to reduce the potential for confounding bias.

However, potential limitations of our study also deserve comment. First, the current study was a cross-sectional study that precludes to establish a temporal relationship between hyperuricemia and knee synovial abnormalities. Further prospective studies are warranted to elucidate the causal relationship. Second, a single measurement of SU concentration may not fully reflect long-term urate status, and we have no knowledge of SU levels before synovial abnormalities developed. Third, although we controlled for several potential confounders, unmeasured confounding cannot be completely ruled out in an observational study.

### Clinical implications

Knee synovial abnormalities are commonly seen in the elder population. Studies have shown that synovial abnormalities are associated with joint pain, swelling, damage, and deformity [3, 6, 42]. Treatment strategies for knee synovial abnormalities are challenging largely because of limited understanding of its pathogenesis. Our study shows that hyperuricemia positively associates with the prevalence, as well as the bilaterality, of knee synovial abnormalities. These findings may shed light on potential mechanisms linking hyperuricemia, a potentially modifiable risk factor, to knee synovial abnormalities and provide a rationale to investigate the effects of urate-lowering treatment on such abnormalities. Further longitudinal studies are required to confirm causality and whether reduction in SU can improve knee synovial abnormalities. In addition, our research further supported that musculoskeletal ultrasound is a viable imaging tool for large-scale epidemiological studies, with economical, readily available, reliable, and therapeutic guiding characteristics [43, 44].

## Conclusions

This population-based study shows that hyperuricemia associates with a higher prevalence and extent of knee synovial abnormalities, suggesting that hyperuricemia, a modifiable risk factor, may play a role in the pathogenesis of knee synovial abnormalities.

### Electronic supplementary material

Below is the link to the electronic supplementary material.

Supplementary Material 1

## Data Availability

The datasets analyzed during the current study are available from the corresponding authors on reasonable request.

## Declarations

### Patient and public involvement

Patients and/or the public were not involved in the design, or conduct, or reporting or dissemination plans of this research.

### Transparency

The lead author affirms that the manuscript is an honest, accurate, and transparent account of the study being reported; that no important aspects of the study have been omitted; and that any discrepancies from the study as planned have been explained.

## Abbreviations

aOR: Adjusted odds ratio
BMI: Body mass index
CI: Confidence interval
EULAR: European League Against Rheumatism
GEE: generalized estimating equations
ICC: Intra-class correlation coefficient
IL-1β: Interleukin-1beta
IL-6: Interleukin-6
MSU: Monosodium urate
OMERACT: Outcome Measures in Rheumatology
OR: Odds ratio
PDS: Power Doppler signal
PPS: probability proportionate to size
RA: Rheumatoid arthritis
SD: standard deviation
SU: Serum urate
TNF-α: Tumor necrosis factor-α
XO Study: Xiangya Osteoarthritis Study
