# Supplementary material for: Associations between hyperuricemia and ultrasound-detected knee synovial abnormalities in middle-aged and older population: a cross-sectional study
Source: J Orthop Surg Res. 2024 Apr 4;19:226. doi: 10.1186/s13018-024-04708-w (PMC10996165; doi:10.1186/s13018-024-04708-w)
Supplement: Supplementary file 1 — Supplementary Material 1 [file 13018_2024_4708_MOESM1_ESM.docx]

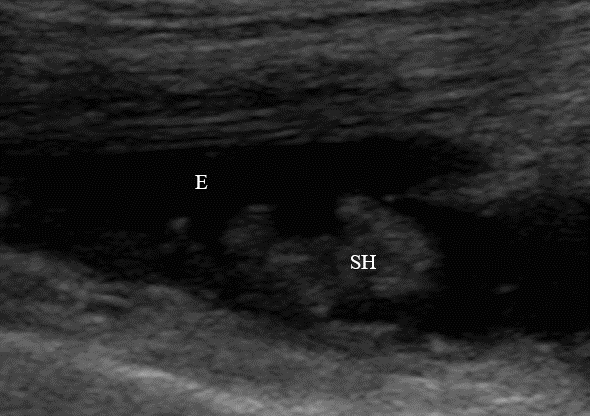


**Supplementary Figure 1. Longitudinal ultrasonographic scan of suprapatellar recess (B mode).** E, joint effusion; SH, synovial hypertrophy.


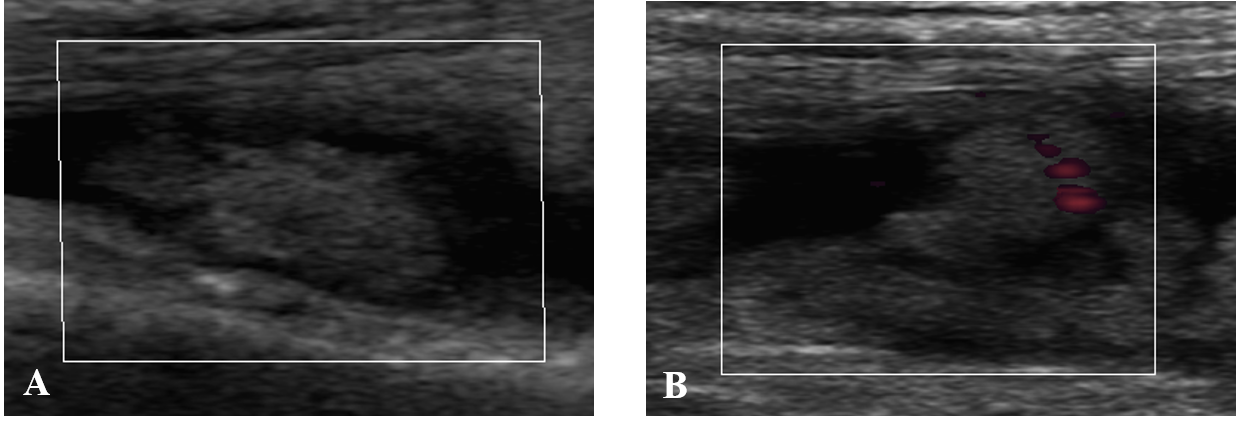


**Supplementary Figure 2. Longitudinal ultrasonographic scan of suprapatellar recess (Power Doppler mode).** Power Doppler signal (PDS) at synovial membrane was scored using a dichotomous scoring system. (A) absent, (B) present.

**Supplementary Table 1. The intra- and inter-rater reliability for ultrasound-detected synovial abnormalities**

|  |  | **Intra-rater reliability** | **Inter-rater reliability** |
| --- | --- | --- | --- |
| **Ultrasonography** | Synovial hypertrophy,  ICC (95% CI) | 0.99 (0.98-1.00) | 0.94 (0.87-0.97) |
|  | Synovial effusion,  ICC (95% CI) | 0.98 (0.92-0.99) | 0.96 (0.93-0.98) |
|  | Power Doppler Signal,  Weighted-Kappa (95% CI) | 1.00(1.00-1.00) | 0.82 (0.66-0.97) |

CI, confidence interval; ICC, intra-class correlation coefficient.

**Supplementary Table 2. Sensitivity analysis of associations between asymptomatic hyperuricemia and knee synovial abnormalities on ultrasound by excluding gout patients**

| **Synovial abnormalities** | **Hyperuricemia** | |
| --- | --- | --- |
|  | **No** | **Yes** |
| **Knee synovial abnormality*** |  |  |
| No, number of knees (%) | 3,735 (65.7) | 676 (64.4) |
| Yes, number of knees (%) | 1,949 (34.3) | 374 (35.6) |
| Crude OR (95% CI) | 1.00 (reference) | 1.06 (0.90, 1.25) |
| Adjusted OR (95% CI)^†^ | 1.00 (reference) | 1.19 (1.00, 1.41) |
| **Synovial hypertrophy** |  |  |
| No, number of knees (%) | 5,098 (89.7) | 906 (86.3) |
| Yes, number of knees (%) | 586 (10.3) | 144 (13.7) |
| Crude OR (95% CI) | 1.00 (reference) | 1.38 (1.10, 1.73) |
| Adjusted OR (95% CI)^†^ | 1.00 (reference) | 1.40 (1.11, 1.78) |
| **Joint effusion** |  |  |
| No, number of knees (%) | 3,851 (67.8) | 695 (66.2) |
| Yes, number of knees (%) | 1,833 (32.2) | 355 (33.8) |
| Crude OR (95% CI) | 1.00 (reference) | 1.07 (0.91, 1.26) |
| Adjusted OR (95% CI)^†^ | 1.00 (reference) | 1.20 (1.01, 1.42) |
| **Power Doppler signal** |  |  |
| No, number of knees (%) | 5,540 (97.5) | 1,009 (96.1) |
| Yes, number of knees (%) | 144 (2.5) | 41 (3.9) |
| Crude OR (95% CI) | 1.00 (reference) | 1.56 (1.05, 2.32) |
| Adjusted OR (95% CI)^†^ | 1.00 (reference) | 1.37 (0.89, 2.10) |

CI, confidence interval; OR, odds ratio.

* Synovial hypertrophy, joint effusion, or Power Doppler signal.

^†^ Adjusted for age, sex, BMI, smoking status, alcohol consumption, educational level, knee injury history, diabetes, hypertension, and serum creatinine.

**Supplementary Table 3. Sensitivity analysis of associations of asymptomatic hyperuricemia and laterality of prevalent of knee synovial abnormalities on ultrasound by excluding gout patients**

| **Synovial abnormalities** | **Hyperuricemia** | |
| --- | --- | --- |
|  | **No** | **Yes** |
| **Knee synovial abnormality*** |  |  |
| No, n (%) | 1,471 (51.8) | 262 (49.9) |
| Unilateral, n (%) | 793 (27.9) | 152 (29.0) |
| Bilateral, n (%) | 578 (20.3) | 111 (21.1) |
| Crude OR (95% CI) | 1.00 (reference) | 1.07 (0.90, 1.27) |
| Adjusted OR (95% CI)^†^ | 1.00 (reference) | 1.28 (1.07, 1.54) |
|  |  |  |
| **Synovial hypertrophy** |  |  |
| No, n (%) | 2,370 (83.4) | 412 (78.5) |
| Unilateral, n (%) | 352 (12.6) | 82 (15.6) |
| Bilateral, n (%) | 114 (4.0) | 31 (5.9) |
| Crude OR (95% CI) | 1.00 (reference) | 1.38 (1.10, 1.74) |
| Adjusted OR (95% CI)^†^ | 1.00 (reference) | 1.41 (1.10, 1.81) |
|  |  |  |
| **Joint effusion** |  |  |
| No, n (%) | 1,532 (53.9) | 272 (51.8) |
| Unilateral, n (%) | 787 (27.7) | 151 (28.8) |
| Bilateral, n (%) | 523 (18.4) | 102 (19.4) |
| Crude OR (95% CI) | 1.00 (reference) | 1.08 (0.91, 1.29) |
| Adjusted OR (95% CI)^†^ | 1.00 (reference) | 1.24 (1.02, 1.50) |
|  |  |  |
| **Power Doppler signal** |  |  |
| No, n (%) | 2,716 (95.6) | 490 (93.3) |
| Unilateral, n (%) | 108 (3.8) | 29 (5.5) |
| Bilateral, n (%) | 18 (0.6) | 6 (1.2) |
| Crude OR (95% CI) | 1.00 (reference) | 1.54 (1.05, 2.27) |
| Adjusted OR (95% CI)^†^ | 1.00 (reference) | 1.35 (0.89, 2.06) |

CI, confidence interval; n, number; OR, odds ratio.

* Synovial hypertrophy, joint effusion, or Power Doppler signal.

^†^ Adjusted for age, sex, BMI, smoking status, alcohol consumption, educational level, knee injury history, diabetes, hypertension, and serum creatinine.
